# Relationship between excessive erythrocytosis and acute mountain sickness: a field study

**DOI:** 10.1186/2054-9369-1-18

**Published:** 2014-08-21

**Authors:** Xiao-Han Ding, Ji-Hang Zhang, Bin Cui, Lan Huang

**Affiliations:** Institute of Cardiovascular Diseases of PLA, Department of Cardiology, Xinqiao Hospital, Third Military Medical University, Chongqing, 400037 China

**Keywords:** Hematology, High altitude, Exposure, Acute mountain sickness

## Abstract

**Background:**

Alterations in hematology, especially erythroid changes, may be involved in acute mountain sickness (AMS) at high altitude. This study aimed to identify the relationship between excessive erythrocytosis and AMS following different durations of high-altitude exposure.

**Methods:**

A total of 692 healthy young Chinese men were recruited for the study in June and July of 2012 and were divided into the following five groups: I) the 24-h group (24 hours after arrival at Lhasa, 3,700 m, *n* = 261); II) the 7-d group (exposed at Lhasa, 3,700 m for seven days, *n* = 99); III) the re-exposure group (re-exposed at Yang Bajing, 4,400 m for seven days after >1 year of acclimation at 3,700 m,*n* = 94); IV) the acclimated group (>1 year of acclimation at 3,700 m, Lhasa, *n* = 42); and V) the sea-level control (control group, Chengdu, *n* = 196). Case report forms were used to record the subjects’ demographic information and AMS-related symptoms. All of the subjects underwent routine blood tests.

**Results:**

The red blood cell (RBC) count fell slightly but was not significant upon acute exposure to high altitude, whereas the hemoglobin concentration ([Hb]) increased significantly. After high-altitude re-exposure, both of the [Hb] and RBC count showed significant increases. The incidence of AMS was 65.1%, 26.3% and 51.1%, respectively in the 24-h, 7-d and re-exposure groups. The [Hb] (*P* = 0.024) and hematocrit (*P* = 0.017) were greater in the AMS^+^ individuals than in the AMS^-^ individuals in 7-d group. A correlation analysis revealed that the [Hb] and hematocrit were closely related with AMS score in 7-d and re-exposure groups, while the RBC showed a correlation with AMS score only in the re-exposure group. The AMS incidence was lowest when the [Hb] was between 140 and 160 g/L in the 24-h and 7-d groups.

**Conclusions:**

AMS is associated with both [Hb] and excessive erythrocytosis. Additionally, our findings indicate the existence of an optimal [Hb] for preventing AMS.

## Background

The hematological system is responsible for the delivery of oxygen and energy as well as the removal of carbon dioxide and the metabolic wastes that have been modified in high altitude hypoxia stress during migrations and among natives of high altitude [1–4]. Excessive erythrocytosis includes increases in the hemoglobin (Hb) and red blood cells (RBC) in the hematological system. The roles of the primary component Hb, in the combination, storage, transport and release of oxygen are of such critical importance that its alteration may be involved in the pathogenesis of AMS in a brief period of time and in acclimation over a longer duration [5, 6]. Additionally, modifications of other parameters and components, such as the RBC, hematocrit (HCT), mean cell volume (MCV), mean corpuscular hemoglobin (MCH) and mean corpuscular hemoglobin concentration (MCHC) in blood, have also been considered to play key roles in the pathophysiological processes in high altitude environments [7].

However, if adaption fails to compensate after arrival at high altitude, a series of symptoms or even acute mountain sickness (AMS) appears. AMS has been considered to be a type of dysfunction of adaption for high altitude that occurs in individuals who have recently arrived at an altitude > 2,500 m [8, 9]. Although it has been studied for hundreds of years, the underlying mechanisms of AMS have not been fully understood [9, 10]. It is known that the hypoxic vasoconstriction and vasodilatation responses are involved in the pathophysiological processes of AMS, however, the hematological alterations have been thought to participate in the pathogenesis of AMS, perhaps due to the delivery of oxygen in the blood flow [11].

The numerous previous studies focused on the hematological alterations in long-term transmigrations, whereas there is scant research on excessive erythrocytosis, especially on Hb, HCT and RBC in short-term exposure to hypoxia and in re-exposure to greater hypoxia and their relationships with AMS [4, 11]. Thus, we postulate that the excessive erythrocytosis that occurs during various high-altitude exposure durations are correlated closely with AMS. Therefore, this present study was performed to explore the roles of excessive erythrocytosis in the pathogenesis of AMS by focusing on the hematological components that relate to the delivery of oxygen, including Hb concentration ([Hb]), RBC, HCT, MCV, MCH and MCHC.

## Methods

### Participants and procedures

The subjects (*n* = 692) were recruited in June and July of 2012 in Chengdu and Lhasa according to the inclusion and exclusion criteria. The inclusion criteria were as follows: healthy males between 18 and 60 years old. The exclusion criteria were people with any of the following conditions: respiratory system diseases, cardiovascular system diseases, neuropsychosis, cerebrovascular diseases, malignant tumors or dysfunctions of the liver or the kidneys. The volunteers were divided into five groups: the acute high-altitude exposure group who ascended to 3,700 m from 500 m in two hours by plane (24-h group, *n* = 261), the 7-d group who was exposed at 4,400 m for 7 days (*n* = 99), the acclimated group who had been acclimated at 3700 m for more than 1 year (*n* = 42) and the re-exposure group who had immigrated to 3,700 m for > 1 year and ascended to a higher altitude of 4,400 m for seven days (*n* = 94). An additional 196 volunteers were selected as sea-level controls.

Subjects who agreed to participate in the study were familiarized with the purposes and processes of this study and signed informed consents before the trials. The study was approved by the Ethics Committee of Xinqiao Hospital, the Second Clinic Medical College of Third Military Medical University.

The participants underwent routine blood tests after a 5 min rest using a BC-3000 plus automated hematology corpuscle analyzer (Shenzhen, China). Structured case report form (CRF) questionnaires were used to record the demographic information and the symptoms of AMS, including the following: headache (0 = without headache; 1 = mild headache; 2 = moderate headache; 3 = severe headache), dizziness (0 = without dizziness; 1 = mild dizziness; 2 = moderate dizziness; 3 = severe dizziness), gastrointestinal symptoms (0 = without and 1 = with gastrointestinal symptoms), insomnia (0 = as well as usual; 1 = not so well as usual; 2 = wake up several times over the night and 3 = difficult to sleep) and fatigue (0 = without fatigue and 1 = with fatigue). AMS was diagnosed by the Lake Louise self-assessment scoring system (LLS) as individuals who arrived at high altitude with headaches and an LLS score >3 [9].

### Statistical analysis

The normally distributed measurement variables [age, body mass index (BMI), [Hb], HCT, RBC, MCV, MCH and MCHC] were expressed as mean ± standard deviation (SD). These variables were employed by independent sample *t* tests between the AMS^+^ and AMS^-^ groups and were compared by ANOVA in the sea-level, 24-h, 7-d, acclimated and re-exposure groups. The relationship between the AMS score and these parameters were analyzed by Pearson’s correlation. The statistical analyses were performed using SPSS 19.0 for Windows. *P* < 0.05 was considered to be statistically significant. Statisticians from the Third Military Medical University were consulted in regard to all of the statistical methods and results.

## Results

The CRFs were excluded if the demographic information was incomplete. A total of 692 valid CRFs were obtained. The ages and BMIs were matched among the five groups (*P* = 0.151 and 0.471, respectively) (Table 1).

**Table 1:** **Demographic information for each group**

Group	Age (year)	BMI (kg/m ^2)^
Sea-level (*n* = 196)	22.23 ± 2.41	21.37 ± 2.44
24-h group (*n* = 261)	22.83 ± 3.65	21.49 ± 2.32
7-d group (*n* = 99)	22.55 ± 3.67	21.52 ± 2.07
Acclimated group (*n* = 42)	24.57 ± 4.81	22.06 ± 2.53
Re-exposure group (*n* = 94)	23.01 ± 2.93	21.08 ± 1.96
*F* value	1.773	0.843
*P* value	0.151	0.471

The ages and BMIs in the five groups were matched.

After exposure at 3,700 m, the [Hb] was elevated significantly compared with that at sea level. The [Hb] increased greatly after the subjects were re-exposed to an altitude higher than 3,700 m. However, the RBC count fell slightly but not significantly after acute exposure, although it increased significantly when the subjects were re-exposed to 4,400 m from their acclimation altitude (3700 m) (Table 2). The HCT was lower in the 24-h group than in the sea-level control group (41.32 ± 3.29 *vs*. 42.71 ± 3.22 L/L, *P* < 0.001), whereas it was higher in the 7-d and re-exposure groups (46.68 ± 3.11 and 53.55 ± 5.84 L/L). However, the MCV was much higher in the re-exposure group than in the other three groups. It is interesting that the re-exposure group was characterized by a lower MCHC than that in the 24-h, 7-d and acclimated groups (*P* = 0.003, *P* < 0.001 and *P* < 0.001), although it was still greater than in the control group (Table 2).

**Table 2:** **Descriptions of the** [**Hb**], **RBC**, **HCT**, **MCV**, **MCH and MCHC for each group**

Group	[Hb] (g/L)	RBC (10 ^12^)	HCT (L/L)	MCV (fl)	MCH (pg)	MCHC (g/L)
Sea-level (*n* = 196)	141.30 ± 10.68	4.66 ± 0.59	42.71 ± 3.22	91.35 ± 7.81	30.37 ± 3.05	331.05 ± 13.78
24-h group (*n* = 261)	145.91 ± 12.94^a^	4.58 ± 0.39	41.32 ± 3.29^a^	90.44 ± 6.44	31.88 ± 2.30	352.39 ± 8.13^a^
7-d group (*n* = 99)	170.92 ± 11.78^ac^	5.15 ± 0.40^ac^	46.68 ± 3.11^ac^	91.01 ± 4.71	33.26 ± 2.12	365.77 ± 9.66^ac^
Acclimated group (*n* = 42)	168.02 ± 1.69 ^ac^	5.06 ± 0.37^ac^	47.05 ± 3.25^ac^	93.12 ± 3.00^b^	33.19 ± 1.20^ac^	356.71 ± 6.01^be^
Re-exposure group (*n* = 94)	185.30 ± 11.53^acef^	5.72 ± 0.61^acef^	53.55 ± 5.84^acef^	93.74 ± 3.98^ace^	32.54 ± 2.40^bd^	347.63 ± 22.60^acef^
*F* value	312.573	112.052	213.735	5.803	30.192	144.797
*P* value^#^	<0.001					

^#^ *P* values among the five groups. a. *P* < 0.01 compared with the sea level group; b. *P* < 0.05, c. *P* < 0.01 compared with the 24-h group; d. *P* < 0.05, e. *P* < 0.01 compared with the 7-d group; f. *P* < 0.01 compared with the acclimated group.

**Figure 1:**
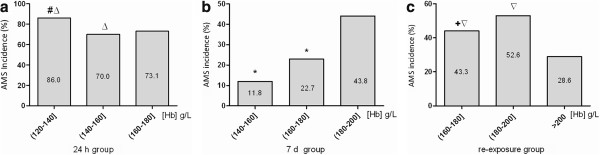
**Comparisons of AMS incidences in variant** **[** **Hb** **]** **spans among the 24**-**h** **,** **7**-**d and re** **-** **exposure groups.** **(a)** 24 -h group, **(b)** 7-d group and **(c)** re-exposure group.

The incidence of AMS was 65.1%, 26.3% and 51.1% in the 24-h, 7-d and re-exposure groups, respectively. Although the subjects in the re-exposure group had been acclimated for more than 1 year, the incidence of AMS was still much higher when they were re-exposed to a new altitude than when they were first exposed for the same duration of time (*χ*^2^ = 12.545, *P* = 0.001). The incidence of AMS was significantly different among a series of spans that were divided by an interval of 20 g/L according to the [Hb] means in each group. The baseline level of the [Hb] increased with the duration of the high-altitude exposure. Thus, the initial [Hb] was inconsistent in the three groups above. The incidences were lowest when the [Hb] was between 140 and 160 g/L in the 24 h and 7-d groups (Figure 1).

The [Hb], RBC, HCT, MCV, MCH and MCHC parameters of the hematological system were not statistically significantly different between the AMS^+^ and AMS^-^ groups in either the 24-h group or the re-exposure group (*P* > 0.05). However, the [Hb] (*P* = 0.024) and HCT (*P* = 0.017) were greater in the AMS^+^ individuals than that in the AMS^-^ ones seven days after their arrival at 4,400 m (Table 3). The [Hb], RBC and HCT each had a negative relationship with AMS score in the 24-h group, but it was not significant for any of them. The [Hb] (*r* = 0.198, *P* = 0.049) and HCT (*r* = 0.207, *P* = 0.039) were significantly positively related with AMS score in the 7-d group, and they were also correlated with AMS score in the re-exposure group (*r* = 0.259, *P* = 0.012 and *r* = 0.213, *P* = 0.040). In addition, the RBC count was also closely associated with the AMS score in the latter group (*r* = 0.239, *P* = 0.020) (Table 4).

**Table 3:** **Differences in the [Hb], RBC, HCT, MCV, MCH and MCHC between the AMS** ^**+**^ **and AMS** ^**-**^ **groups**

	24-h group				7-d group				Re-exposure group			
	AMS ^+^ *(n* = 190)	AMS ^-^ *(n* = 71)	*t*	*P*	AMS ^+^ ( *n* = 26)	AMS ^-^( *n* = 73)	*t*	*P*	AMS ^+^ ( *n* = 48)	AMS ^-^( *n* = 46)	*t*	*P*
[Hb] (g/L)	145.77 ± 12.04	146.55 ± 15.24	-0.432	0.666	175.38 ± 10.82	169.33 ± 11.77	2.299	0.024^*^	187.06 ± 12.69	183.46 ± 9.99	-1.527	0.130
RBC (10^12^)	4.57 ± 0.39	4.62 ± 0.40	-0.990	0.323	5.23 ± 0.35	5.12 ± 0.41	1.225	0.223	5.81 ± 0.72	5.62 ± 0.45	-1.519	0.132
HCT (L/L)	41.29 ± 3.08	41.48 ± 3.83	-0.398	0.691	47.91 ± 2.69	46.24 ± 3.15	2.419	0.017^*^	54.34 ± 6.93	52.72 ± 4.36	-1.358	0.178
MCV (fl)	90.77 ± 5.48	89.58 ± 8.49	1.331	0.184	91.87 ± 2.81	90.71 ± 5.21	1.085	0.281	93.64 ± 4.30	93.86 ± 3.66	0.268	0.789
MCH (pg)	31.96 ± 2.19	31.70 ± 2.59	0.797	0.426	33.54 ± 1.23	33.16 ± 2.36	0.777	0.439	32.39 ± 2.66	32.69 ± 2.11	0.600	0.550
MCHC (g/L)	352.34 ± 7.60	352.51 ± 9.48	-0.144	0.886	365.50 ± 5.53	365.86 ± 10.79	-0.164	0.870	346.52 ± 25.32	348.78 ± 19.57	0.483	0.630

*P < 0.05.

**Table 4:** **Relationships between the AMS score and the** [**Hb**], **RBC**, **HCT**, **MCV**, **MCH and MCHC**

Index	24-h group		7-d group		Re-exposure group	
	Pearson’s *r*value	*P*value	Pearson’s *r*value	*P*value	Pearson’s *r*value	*P*value
[Hb]	-0.065	0.297	0.198^*^	0.049	0.259^*^	0.012
RBC	-0.076	0.222	0.192	0.057	0.239^*^	0.020
HCT	-0.069	0.264	0.207^*^	0.039	0.213^*^	0.040
MCV	0.056	0.372	-0.015	0.885	-0.050	0.632
MCH	0.024	0.694	-0.018	0.859	-0.091	0.385
MCHC	-0.027	0.667	-0.021	0.833	-0.064	0.538

*P < 0.05.

## Discussion

The hematological components related to the delivery of oxygen were affected differently after high altitude exposure. The [Hb] responded more rapidly to short-term hypoxia than RBC in that it was related closely with AMS in both the 7-d group and re-exposure group. The incidences were lowest when the [Hb] was between 140 g/L and 160 g/L, which generally indicates an optimal [Hb] level.

### Excessive erythrocytosis after high-altitude exposure

The [Hb] modified sharply after elevations to 3,700 m, and it rose gradually along with increases in the duration of the exposure, which was consistent with previous research on acclimated populations [12, 13]. However, more RBC in the blood after a longer duration of acclimation may be caused by the increased erythropoietin (EPO) level that corresponds to hypobaric hypoxia in such a chronic progressive process [4, 14]. Thus, it can be concluded that the alterations in Hb occur prior to changes in the RBC. We also observed that the HCT had a more extensive elevation in the 24-h group, while the RBC count had a slight reduction, which would indicate that the individuals had suffered transient hemodilution upon acute high-altitude exposure, which was conflicted with the view that acute pachyhemia occurs upon exposure to high altitude. Nevertheless, the re-exposure group had the highest RBC count, which was in agreement with several studies on migration and natives of high altitude [1, 3, 4, 15].

There was no significant difference in the volume of the RBC in the former three groups except that the increased MCV in the re-exposure group showed a modification of the RBC in the long-term hypoxia environment. Thus, the augmentation of MCHC can be ascribed to the augmented [Hb] combined with the unchanged MCV.

### The relationships between AMS and excessive erythrocytosis

The conflicts among the alterations of Hb, HCT and RBC may be potential mechanisms of AMS.

The [Hb], RBC, HCT, MCV, MCH and MCHC were not significantly different between the AMS^+^ and AMS^-^ populations upon acute high altitude stress. However, in the 7-d group, the [Hb] and HCT levels were higher in the AMS^+^ individuals than that in the AMS^-^ ones. We have observed that the [Hb] and HCT were related to the AMS scores in both the 7-d and re-exposure groups, and the RBC count was also related to AMS in the latter group. Those results revealed that the rapid response of Hb and lengthier alteration in RBC would be critical pathogenesis processes of AMS.

The alterations of [Hb], HCT and RBC alter the hemorheology and hemodynamics. One mechanism of AMS is that the greater augmentations of HCT and RBC elevate the viscosity of the blood and decrease the cardiac output, which reduces the delivery of oxygen [2].

There was a novel interesting discovery that the RBC count increased rapidly upon the subjects’ exposure to a higher altitude. This observation reveals that long-term hypoxia may condition the hematopoietic system to be more sensitive to more extended hypoxia and that the damage induced by hypoxia had already occurred. The mechanisms of the above-mentioned phenomenon perhaps are related to the over-expressed and augmented EPO and EPO receptors resulting from persistent hypoxia. These alterations in the EPO and EPO receptors effectually enhance hematopoiesis, although the erythrocythemia had not yet emerged [14, 16].

### Is there an optimal [Hb] for the prevention of AMS?

The elevations in the [Hb], RBC and HCT after migrations to high altitude had been described decades ago and were thought to be advantageous compensations [3, 5]. However, the benefits of excessively increased Hb, RBC and HCT and even the pathological states, such as high altitude polycythemia (HAPC), have been challenged by the side effects of the enhanced viscosity and the slowed blood flow velocity and cardiac output resulting in the decreased delivery of oxygen to the tissues [12]. Additionally, there was also a theoretical conclusion that the optimal [Hb] for the human body for adaption to high altitude is 147.0 g/L [12].

Although the optimal [Hb] has been demonstrated by a theoretical study, few practical clinical investigations have been performed to confirm the precise value of the so-called optimal [Hb] [12].

In our current study, the lowest incidence of AMS fall within the span of 140 to 160 g/L, which is consistent with the theoretical approximate value of an optimal [Hb] of 147.0 g/L [12]. Thus, the excessive increases in the Hb and RBC are not entirely beneficial for the human body’s acclimation to high altitude. The influence of the variations in the participants’ sizes on the incidence of AMS cannot be overlooked in the re-exposure group when the [Hb] was greater than 200 g/L.

### Limitations

There were only young men in our study, which perhaps induced an age and gender bias, which will be adjusted in future studies.

## Conclusions

The changes in the [Hb], HCT and RBC counts were not isochronous after high-altitude exposures. Our observations indicate that AMS is associated with Hb and that excessive erythrocytosis may facilitate the pathogenesis of AMS. Additionally, our findings indicate the existence of an optimal [Hb] for preventing AMS.

## Abbreviations

AMS: Acute mountain sickness
Hb: Hemoglobin
[Hb]: Hemoglobin concentration
RBC: Red blood cell
MCV: Mean cell volume
MCH: Mean corpuscular hemoglobin
MCHC: Mean corpuscular hemoglobin concentration
CRF: Case report form
BMI: Body mass index
EPO: Erythropoietin
HAPC: High altitude polycythemia.
